# Diosmin Alleviates Doxorubicin-Induced Liver Injury via Modulation of Oxidative Stress-Mediated Hepatic Inflammation and Apoptosis via NfkB and MAPK Pathway: A Preclinical Study

**DOI:** 10.3390/antiox10121998

**Published:** 2021-12-15

**Authors:** Abdullah F. AlAsmari, Metab Alharbi, Faleh Alqahtani, Fawaz Alasmari, Mohammed AlSwayyed, Sami I. Alzarea, Ibrahim A. Al-Alallah, Adel Alghamdi, Hassan M. Hakami, Meshal K. Alyousef, Youssef Sari, Nemat Ali

**Affiliations:** 1 Department of Pharmacology and Toxicology, College of Pharmacy, King Saud University, P.O. Box 55760, Riyadh 11451, Saudi Arabia; mesalharbi@ksu.edu.sa (M.A.); afaleh@ksu.edu.sa (F.A.); ffalasmari@ksu.edu.sa (F.A.); 441105941@student.ksu.edu.sa (A.A.); 437103103@student.ksu.edu.sa (H.M.H.); 437104079@student.ksu.edu.sa (M.K.A.); 2 Department of Pathology, College of Medicine, King Saud University, Riyadh 11451, Saudi Arabia; malswayyed@ksu.edu.sa; 3 Department of Pharmacology, College of Pharmacy, Jouf University, Sakaka 72341, Saudi Arabia; samisz@ju.edu.sa; 4 Pathology and Clinical Laboratories Medicine, King Fahad Medical City, Riyadh 11451, Saudi Arabia; ialallah@kfmc.med.sa; 5 Department of Pharmacology and Experimental Therapeutics, College of Pharmacy and Pharmaceutical Sciences, University of Toledo, Toledo, OH 43606, USA; youssef.sari@utoledo.edu

**Keywords:** diosmin, doxorubicin, hepatotoxicity, oxidative stress, inflammation, apoptosis

## Abstract

Hepatotoxicity caused by chemotherapeutic drugs (e.g., doxorubicin) is of critical concern in cancer therapy. This study focused on investigating the modulatory effects of diosmin against doxorubicin-induced hepatotoxicity in Male Wistar rats. Male Wistar rats were randomly divided into four groups: Group I was served as control, Group II was treated with doxorubicin (20 mg/kg, intraperitoneal, i.p.), Group III was treated with a combination of doxorubicin and low-dose diosmin (100 mg/kg orally), and Group IV was treated with a combination of doxorubicin and high-dose diosmin (200 mg/kg orally) supplementation. A single dose of doxorubicin (i.p.) caused hepatic impairment, as shown by increases in the concentrations of serum alanine aminotransferase, aspartate aminotransferase, and alkaline phosphatase. Doxorubicin produced histological abnormalities in the liver. In addition, a single injection of doxorubicin increased lipid peroxidation and reduced glutathione, catalase, and superoxide dismutase (SOD) levels. Importantly, pre-treatment with diosmin restored hepatic antioxidant factors and serum enzymatic activities and reduced the inflammatory and apoptotic-mediated proteins and genes. These findings demonstrate that diosmin has a protective effect against doxorubicin-induced hepatotoxicity.

## 1. Introduction

Doxorubicin (DOX) is an anticancer agent and is considered an effective anthracycline antibiotic. It has been effective in both solid and hematological cancers [1,2]. Nevertheless, DOX could negatively affect non-cancer cells; therefore, its clinical practice is limited. In the context of its mechanical action, DOX has more capabilities to target cancer cell growth and inhibit free radical production and DNA intercalation, as shown in in-vitro and in-vivo systems [3,4]. It can induce toxicities, as shown by redox signaling on mitochondria and drastic generation of superoxide radicals and ROS, causing oxidative stress [5,6]. The liver has been studied as a preeminent metabolic organ for DOX and has been considered the most susceptible organ for DOX-induced injury and oxidative stress. DOX treatment can cause varying degrees of hepatic damage in cancer patients [7,8,9].

It has been evidenced that some toxicities, including hepatotoxicity triggered by DOX, could not be reversed. Hepatotoxicity’s detrimental consequence has been associated with anticancer agents [10]. The precise episodes of DOX-provoked toxicity remain debatable. The oxidative machinery has long been recognized for its apoptotic cascade. Apoptosis or programmed cell death is important for proper functioning and cellular survival. Oxidative stress and apoptotic cascade are intently associated with a physiological episode and implicated in various toxicities, including hepatotoxicity [11].

In an oxidative stress-mediated apoptotic event, mitogen-activated protein kinases (MAPKs) are critical for apoptotic signaling [12,13]. MAPKs, known as serine/threonine kinases, can phosphorylate their specific substrates upon stimulation [14]. These phosphorylation cascades could be physiological or pathological, which regulate the phosphorylated substrate, causing integral signaling cascade activities. Therefore, MAPK signaling pathways may modulate gene expression and apoptotic cascades [15,16]. MAPK family consists of three major kinase proteins, ERK, JNK, and p38, which are closely linked to cell growth and differentiation. Interestingly, these proteins are associated with inflammation, apoptosis, and cell death. p38 MAPK has also been involved in apoptotic events by stimulating cellular mortality [17,18]. In addition, p38 MAPK could also mediate several cellular programs, including cell proliferation, differentiation, and cell death in response to different stimuli [19]. Akt, also known as protein kinase B, is another type of kinase protein that has been implicated in phosphorylation, which is activated via extracellular factors involving phosphoinositide 3-kinase (PI3K)-dependent events. Akt is a crucial regulator and/or inhibitor for apoptosis and survival. Evidence has shown that chemotherapeutic intervention is critically linked to reactive oxygen species (ROS) production, which activates p38 and inactivates pAKT signaling, leading to apoptosis in rat tissues [20,21]. Xenobiotic or drug-induced hepatotoxicity showed a generation of free radicals and formation of ROS that led to oxidative stress in different organs [22]. In recent years, inflammation has received considerable attention in toxicities, including haptato toxicity.

DOX-induced hepatoxicity has been associated with the induction of inflammatory response, which could be decreased by reducing the expression of nuclear factor κB (NF-κB). DOX triggers ROS, which plays a crucial role in anti-cancer signaling events, including tumor suppressor p53 and cytochrome-c release, followed by activating caspase enzymes and causing apoptosis [23]. Consequently, concurrent interference of oxidative stress, inflammation, and apoptosis must be a potent strategy to target DOX-induced hepatotoxicity.

In particular, numerous hepatoprotective medications have been ubiquitously employed to determine fetotoxicity [24]. Flavonoids are promising ubiquitous groups of the phenolic plant [25]. They are considered active nutraceutical ingredients in plants. Flavonoids have been demonstrated to have antioxidant [26], anti-inflammatory [27], anti-apoptotic [28], hepatoprotective [10], antiviral [29], and antitumorigenic [30] activities. Diosmin protects against injury of certain organs, including hepatic injury [31,32]. These studies suggest that diosmin has hepatoprotective effects against different toxicants. Nevertheless, to the best of our knowledge, no report has illustrated its protective properties against DOX-induced hepatic damage. Accordingly, our study was designed to examine the effects of diosmin on DOX-induced hepatoxicity and determine the oxidative stress markers and inflammatory, apoptotic signaling pathways involving the protective effects of diosmin.

## 2. Material and Methods

### 2.1. Animals

Male Wistar rats were procured from the animal Maintenance house at the College of Pharmacy, King Saud University (KSU), Riyadh, Saudi Arabia. Animals were maintained at approved standardized conditions. KSU Local Institutional Study Ethics Committee (REC) approved all animal treatments and procedures (approval # KSU-SE-19-121).

### 2.2. Experimental Design

In the present study, 32 male Wistar rats weighing 180–200 g (*n* = 8) were tested and divided into four groups. Rats were acclimated to the vivarium for one week before the initiation of the study. Group I rats were administered with an oral formulation of normal saline and considered as the vehicle group. In Group II, rats were exposed to DOX (20 mg/kg i.p., single dose) on Day 17, as described previously [33]. Group III and Group IV rats were prophylactically administered 100 and 200 mg/kg of diosmin (AK Scientific, Cat# J99039, Union City, CA, USA) via oral gavage, respectively, for the first 18 days and then exposed to DOX (20 mg/kg, i.p.) on day 17, as Group II rats. Rats were euthanized with ketamine/xylazine solution. Blood was withdrawn through the heart ventricle, and liver lobes were isolated and immediately placed into liquid nitrogen. The frozen tissues were used for biochemical and protein expression analysis. Other liver lobes were rinsed in PBS and immediately post-fixed in 4% formaldehyde solution for histological analysis. Mortality was not observed during the entire research protocol in any experimental animal group.

### 2.3. Determination of Serum Hepatotoxicity Markers

Blood samples were withdrawn from the heart ventricles during euthanasia with isoflurane. For serum separation, blood samples were centrifuged for 10 min at 5000× *g* at 4 °C. Collected serum samples were analyzed to quantify liver marker enzymes found in the blood, such as alanine aminotransferase (ALT), aspartate aminotransferase (AST), and alkaline phosphatase (ALP). These were analyzed using a biochemistry autoanalyzer (Dimension^®^ RXL MAXTM, Siemens, Malvern, PA, USA.)

### 2.4. Determination of Lipid Peroxidation

Hepatic tissues were selected to measure lipid peroxidation (LPO), as described in the previous study [34]. In brief, the homogenates were mixed with thiobarbituric acid (TBA) and trichloroacetic acid (TCA), followed by incubation in the water bath, with shaking at 95 °C for 30 min. Subsequently, liver samples were kept on ice for 10 min and centrifuged at 5000× *g* rpm for 15 min at 4 °C. The absorbance values were obtained at 540 nm. Readings were indicated as nmol of malondialdehyde (MDA) formed per mg of protein.

### 2.5. Quantification of Reduced Glutathione

Glutathione (GSH) contents in hepatocytic post-mitochondrial supernatants (PMS) were quantified as described previously [35]. PMS was obtained following Tahir et al. [32]. In brief, Bis (3-carboxy-4-nitrophenyl) disulfide was mixed with PBS in a cuvette to acquire the absorbance values at 412 nm. The quantification of GSH was expressed in nmol/mg protein.

### 2.6. Determination of Catalase Activity

After centrifugation of hepatic homogenates, PMS were collected to measure the activity of catalase (CAT) as described previously [36]. Concisely, the reaction blend contained 1950 μL (100 mM, pH 7.4) of phosphate buffer, 1000 μL (19 mM) of hydrogen peroxide, and 50 μL of PMS, achieving 3 mL (total) in a cuvette. The kinetic was quantified for 5 min at 240 nm. The difference between initial and final values was calculated as CAT activity in the processed samples. The analyzed values were depicted as moles of H_2_O_2_ changed/min/mg protein.

### 2.7. Immunoblot Analysis

Immunoblot analysis was carried out as described previously [37]. Hepatic tissues were lysed, proteins were extracted, and equivalent amounts of proteins (0.02–0.05 mg) from control and treatment groups were loaded into 10–12% of SDS-PAGE for electrophoresis, followed by transfer to PVDF membranes. The membranes were then blocked with 5% milk (non-fat dried) for 60 min and incubated with primary antibodies, such as Bax, Bcl-2, cleaved caspase-3, p65-NFkB, SOD, pAKT, tAkt, P-p38, tp38, and GAPDH, overnight at 4 °C. Membranes were washed and incubated with HRP conjugated specific secondary antibodies for 60 min at room temperature. Proteins were visualized on the membrane using an ECL reagent kit, and Western blot images were captured and analyzed using an instrument for gel imaging (Hercules, CA, Bio-Rad-USA).

### 2.8. Gene Expression Studies (RT-qPCR)

We isolated total RNA from liver tissues using TRIzol™ reagent (Thermo Scientific, USA) as per the manufacturer’s instructions. Purity and concentrations of the isolated RNA samples were measured using a NanoDrop™ 8000 Spectrophotometer (Thermo Scientific, USA). After that, we synthesized cDNA from the isolated RNA using a All-in-One cDNA Synthesis SuperMix (Bimake, Houston, TX, USA). Then, differences in the expression of different genes were quantified using Applied Biosystems 7500 Fast Real-Time PCR System using SYBR green master mix (Bimake, Houston, TX, USA). GAPDH was used as the housekeeping gene. Then, the ΔΔCt method were used to calculate the relative expression of different genes. Sequences of the used primers (IDT, Leuven, Belgium) are described in Table 1.

### 2.9. Histopathology Studies

Hepatic tissues were post-fixed in 4% formaldehyde and then prepared for paraffin sectioning. Paraffin sections were cut at 3 μm using a microtome. Wax-embedded sections were then processed, and wax was removed. The unwaxed hepatic sections were then stained with dyes, hematoxylin, and eosin (H & E), and hepatic histological images were captured using a DP72 camera attached to an Olympus B.X. microscope (Melville, FL, USA).

### 2.10. Statistical Analysis

Data were analyzed using computer-based software graph pad prism 5 (San Diego, CA, USA) and expressed as mean ± S.D. Changes were estimated using one-way ANOVA followed by Tukey’s comparison test, with *p* < 0.05 being statistically significant.

## 3. Results

### 3.1. Effect of Diosmin against DOX-Induced Hepatic Injury

**Figure 1:**
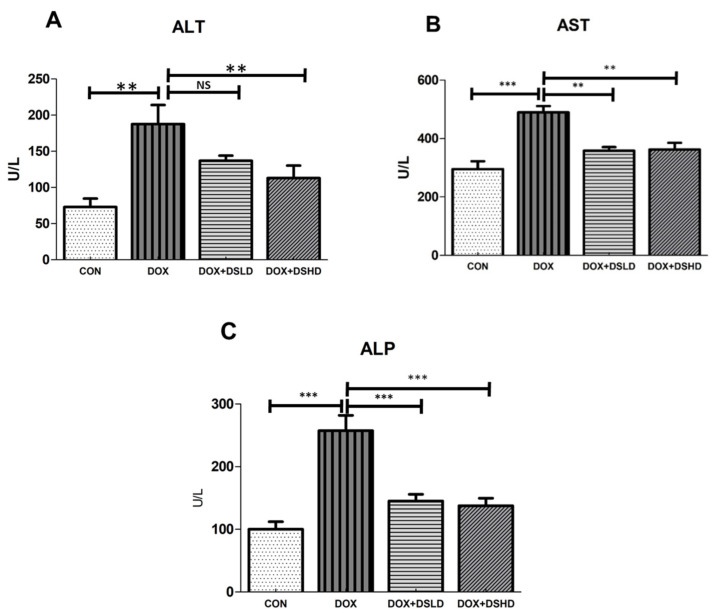
Effects of diosmin pretreatment on serum levels of ALT (**A**), AST (**B**), and ALP (**C**) in male Wistar rats exposed to DOX. DOX increased ALT, AST, and ALP, and diosmin attenuated these effects at both lower and higher dose. Error bars represent the mean ± SD (*n* = 5), where *** *p* < 0.001, ** *p* < 0.01, and NS *p* > 0.05. CON, control group; DOX, doxorubicin group; DOX + DSLD, group of doxorubicin and diosmin 100 mg/kg; DOX + DSHD, group of doxorubicin and diosmin 200 mg/kg.

Evidence shows the serum markers are an influential tool to diagnose the hepatic impairment; therefore, it become essential to measure the serum markers as indicators for hepatotoxicity. To examine whether diosmin supplementation prevents DOX-induced alteration in hepatic functions, we measured blood serum concentrations of ALT, AST, and ALP. We revealed that a single injection of DOX dose induced hepatic injury, as evidenced by the increase in serum ALT, AST, and ALP concentrations (Figure 1A–C). Nevertheless, diosmin pretreatment attenuated DOX-induced increase in concentrations of these markers to the normal levels.

### 3.2. Effects of Diosmin against DOX-Induced Oxidative Stress Markers and Inactivation of Enzymatic Antioxidants

**Figure 2:**
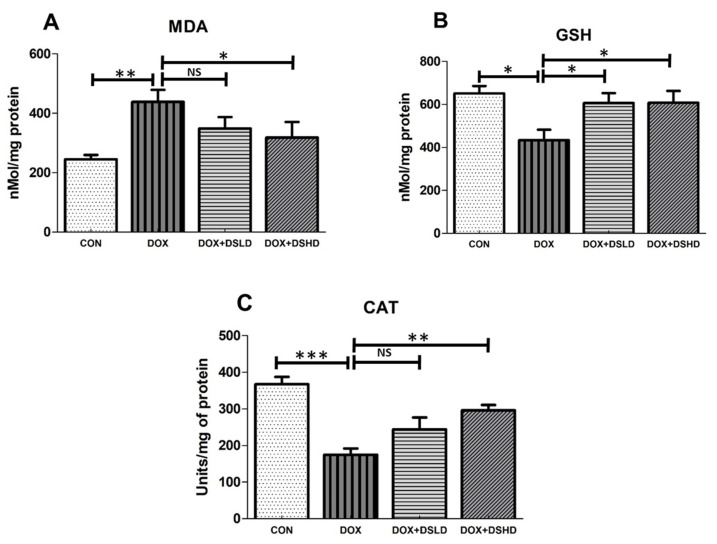
Effects of diosmin pretreatment on oxidative stress markers, such as MDA (**A**), GSH (**B**), and CAT (**C**), in male Wistar rats exposed to DOX. Diosmin diminished the oxidative stress induced by DOX. DOX increased MDA and decreased GSH and CAT, and diosmin attenuated these effects at both lower and higher dose. Error bars represent the mean ± SD, where *** *p* < 0.001, ** *p* < 0.01, * *p* < 0.05, and NS *p* > 0.05. CON, control group; DOX, doxorubicin group; DOX + DSLD, group of doxorubicin and diosmin 100 mg/kg; DOX + DSHD, group of doxorubicin and diosmin 200 mg/kg.

Nutraceuticals have been implicated as therapeutic molecules modulating several kinds of oxidative stress-mediated toxicity, including hepatotoxicity [11]. To further explore the protective effect of diosmin, hepatic tissue MDA levels, GSH, and CAT activity were determined in hepatocytes. The findings revealed that DOX (20 mg/kg, single dose) increased tissue MDA levels and decreased GSH levels and enzymatic activities of CAT. Nevertheless, diosmin prophylactic treatment significantly restored LPO, GSH, and CAT catalytic activity in a dose-dependent manner. These findings revealed the protective effect of diosmin involving free radical scavenging (Figure 2A–C).

### 3.3. Effect of Diosmin against DOX-Induced Changes in Oxidative Stress, Inflammatory Markers, and MAPK Pathway

**Figure 3:**
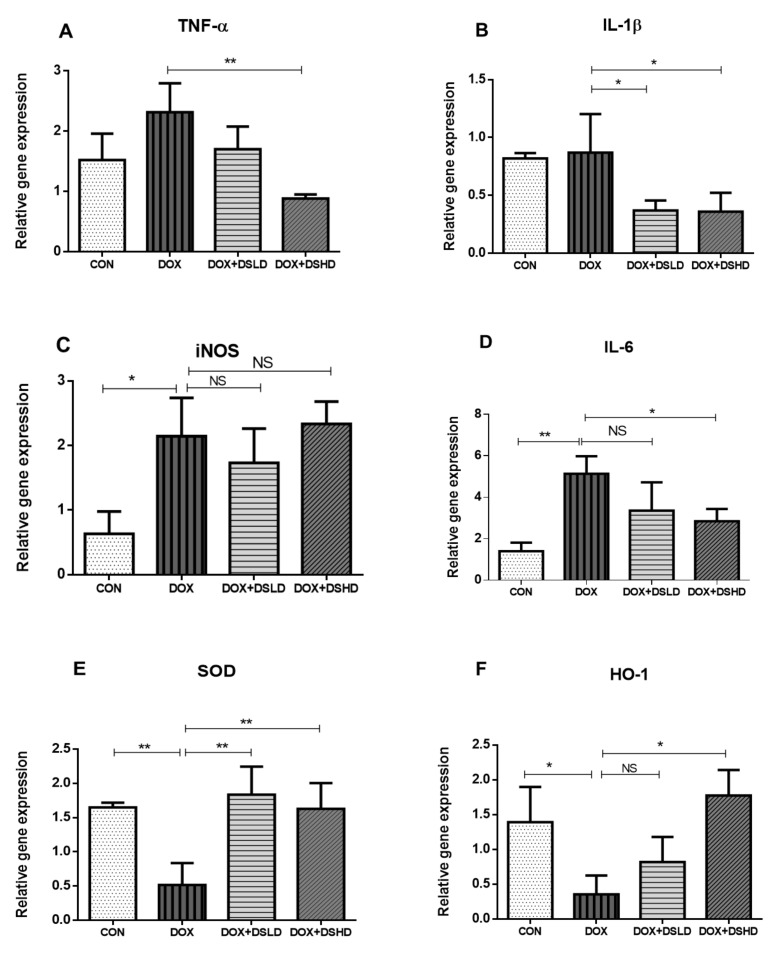
(**A**–**F**) Effects of diosmin pretreatment on the gene expression of inflammatory and antioxidants genes. Data are presented as mean ± SD (*n* = 3), where ** *p* < 0.01, * *p* < 0.05 and NS *p* > 0.05. CON, control group; DOX, doxorubicin group; DOX + DSLD, group of doxorubicin and diosmin 100 mg/kg; DOX + DSHD, group of doxorubicin and diosmin 200 mg/kg.

**Figure 4:**
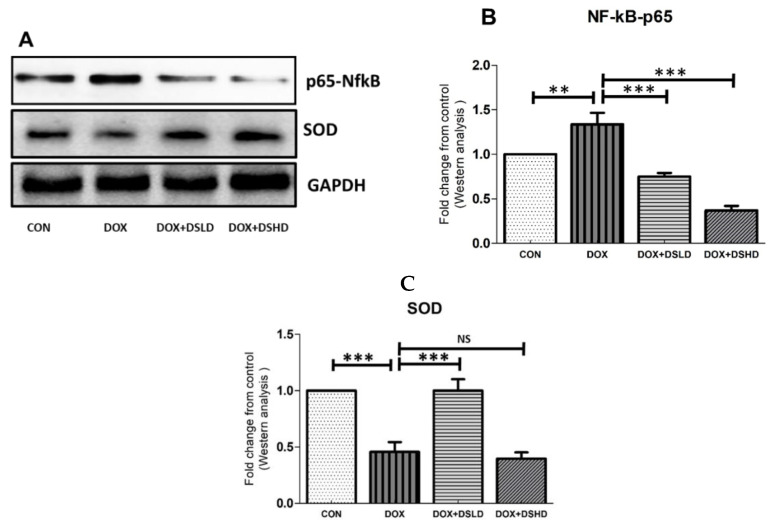
Effects of diosmin pretreatment altered the level of protein expression of p65-NFkB and SOD. (**A**) Immunoblot representation of p65-NFkB and SOD; (**B**,**C**) graphical representation of p65-NFkB and SOD. Error bars represent the mean ± SD (*n* = 3). *** *p* < 0.001, ** *p* < 0.01, and NS *p* > 0.05. CON, control group; DOX, doxorubicin group; DOX + DSLD, group of doxorubicin and diosmin 100 mg/kg; DOX + DSHD, group of doxorubicin and diosmin 200 mg/kg.

**Figure 5:**
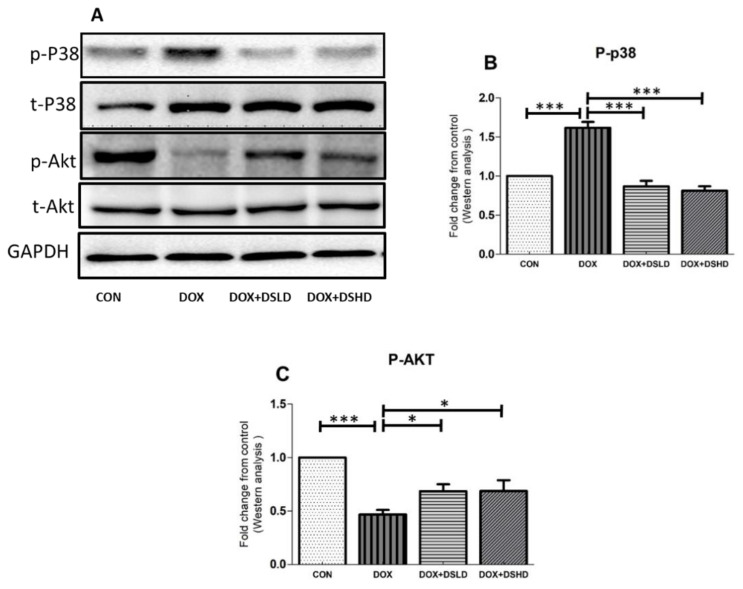
Effects of diosmin pretreatment on altered the level of p38- and pAKT-mediated apoptosis cascade. (**A**) Immunoblot representation of p38 and pAKT; (**B**,**C**) graphical representation of p38 and pAKT. Error bars represent the mean ± SD (*n* = 3). *** *p* < 0.001 and * *p* < 0.05. CON, control group; DOX, doxorubicin group; DOX + DSLD, group of doxorubicin and diosmin 100 mg/kg; DOX + DSHD, group of doxorubicin and diosmin 200 mg/kg.

Particular attention has been devoted toward chemotherapeutics that could inhibit and suppress oxidative stress trafficking MAPK pathways. Such kinds of events could also be restored by nutraceuticals. In addition, DOX treatment inhibited PI3K/AKT pathway, consistent with the previous findings. The antioxidative and anti-inflammatory activities of diosmin have been demonstrated in a previous study [38]. Therefore, we evaluated oxygen-free radical scavenging and anti-inflammatory potential of diosmin by measuring the gene and protein expression of different oxidative stress and anti-inflammatory markers. As expected, DOX treatment increased the gene expression of iNOS, TNF-a, IL-1b, and IL-6 compared to control group (Figure 3A–D). Furthermore, the gene expressions of HO-1 and SOD were considerably reduced in DOX-treated animals (Figure 3E,F). Nonetheless, diosmin pre-treatment mitigated these alterations induced in response to DOX exposure in a dose-dependent manner (Figure 3A–F). DOX treatment drastically reduced the expression of SOD and pAKT, while it significantly enhanced protein expression of NfkB-p65 and P-p38 (Figure 4A–C and Figure 5A–C). Interestingly, diosmin pre-treatment alleviated DOX-induced alterations in all the proteins mentioned earlier (Figure 4A–C and Figure 5A–C). Diosmin might maintain an equilibrium balance of antioxidant status in SOD, restoring NfkB-p65, p-pAKT, and p-p38 signaling. These results further substantiate the potent antioxidant and anti-inflammatory effects of diosmin on DOX administration.

### 3.4. Effect of Diosmin against DOX-Induced Activation of Apoptotic Pathways

**Figure 6:**
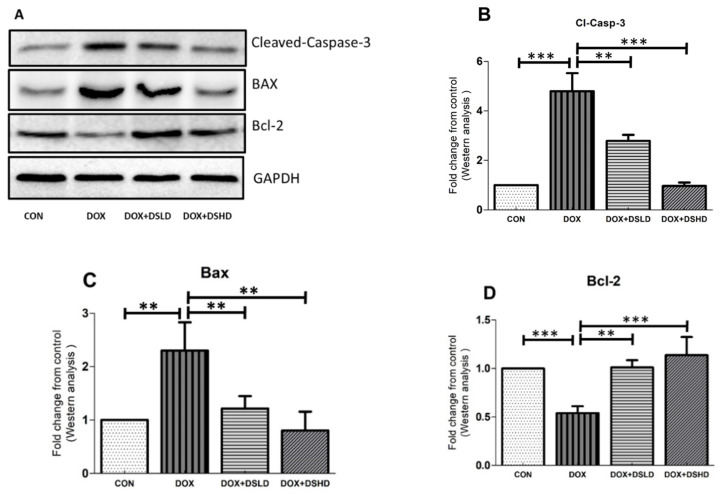
Effects of diosmin pretreatment on the expression of pro- and antiapoptotic protein and cleavage action of caspase in apoptosis cascade. (**A**) Immunoblot representation of Cleaved Caspase 3, BAX, and Bcl-2; (**B**–**D**) graphical representation of Cleaved Caspase 3, BAX, and Bcl-2. Error bars represent the mean ± SD (*n* = 3). *** *p* < 0.001 and ** *p* < 0.01. CON, control group; DOX, doxorubicin group; DOX + DSLD, group of doxorubicin and diosmin 100 mg/kg; DOX + DSHD, group of doxorubicin and diosmin 200 mg/kg.

Predominantly, anticancer compounds were investigated under in-vivo and clinical trials to confirm the full spectrum of toxicity, including hepatotoxicity [11]. In addition, several reports have proposed that diosmin might instantly rescue oxidative stress-induced toxicity and activate the apoptotic cascade in cells or tissues. Previous studies have demonstrated that diosmin possesses anti-apoptotic properties [39]. Therefore, to further scrutinize diosmin against DOX-hepatotoxicity, we evaluated protein expression of different apoptotic proteins. As anticipated, single injection of DOX caused a significant increase of both cleaved caspase-3 and Bax (Figure 6A–D). In contrast, DOX exposure reduced the expression of anti-apoptotic protein Bcl-2 as compared to the control group I. Diosmin pre-treatment, however, restored the expression of these marker proteins (Figure 6A–D). These data indicate that diosmin pre-supplementation is critical in the attenuation of apoptosis induced by DOX in the liver.

### 3.5. Effect of Diosmin against DOX-Induced Hepatic Histological Alterations

**Figure 7:**
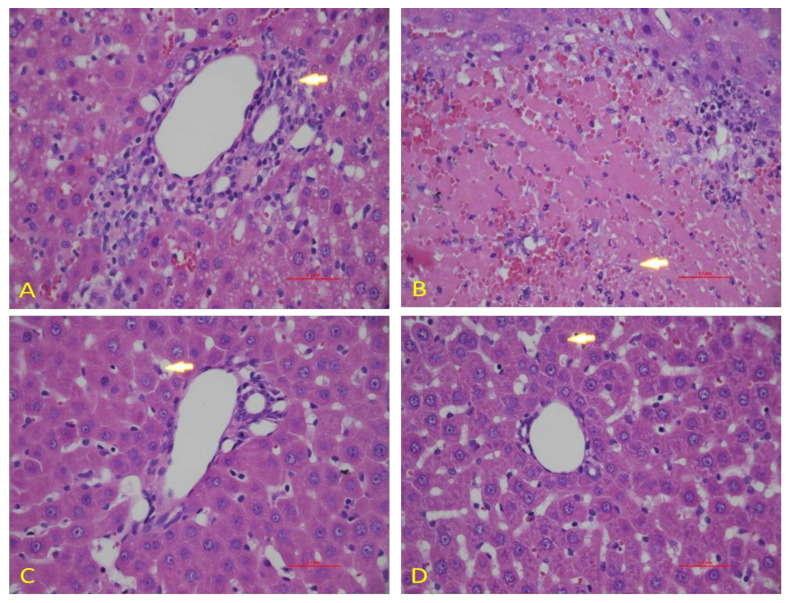
Effects of diosmin pretreatment on light micrographs of the H & E stain liver tissues of 0.1 mm. (**A**) The normal tissue of the liver. (**B**) Irregular architecture of liver tissues caused by Dox administration appeared, as indicated by arrows. (**C**,**D**) Diosmin pre-treatment reduced harmful effects of Dox in cytoplasmic vacuoles, necrotic hepatocytes, and vascular congestion at both doses.

To confirm the data obtained in our biochemical studies, we evaluated the histological architecture of hepatic tissues after exposure to DOX. We observed a normal hepatic architecture in control animals (Figure 7A). However, DOX exposure caused significant damage in hepatic cells, as shown by the induction of hepatocytes necrosis with marked neutrophilic infiltration (Figure 7B). Nevertheless, diosmin pretreatment showed the presence of a control vein with surrounding normal hepatocytes. However, few residual necrotic hepatocytes were observed in a dose-dependent manner (Figure 7C,D).

## 4. Discussion

Doxorubicin (DOX) is an effective anthracycline antibiotic to treat both solid and hematological cancers. Nevertheless, DOX could negatively affect non-cancer cells; therefore, its clinical practice is limited [1,2]. It has been shown that DOX-induced oxidative damage is associated with acute toxicity that induced by single dose of DOX in animals [40,41]. Furthermore, it has been reported that DOX toxicity can be divided into three categories: acute, subacute, and chronic toxicity [42,43]. Acute toxicity (short-term model) is induced following a single dose of DOX (dose usually ranges from approximately 5–30 mg/kg) and can lead to liver, renal, and cardiac damage. The long-term model (chronic toxicity of DOX), however, is induced after using multiple low doses of DOX over the period of 2–12 weeks [42,43]. Therefore, in the current study, we used a single dose of DOX to induce acute liver damage according to previous studies. Furthermore, the single dose of DOX that we have used in the current study (20 mg/kg) is corresponding to a high single dose in the clinic for treating cancer patients [42].

Serum transaminases may be contemplated as a perceptive index of hepatic injury [44]. Hepatic impairment and/or injury alter their transport machinery and modulate membrane permeability. This could lead to the flow of certain specialized enzymes from hepatic cells, resulting in a minimal range of ALT, AST, and ALP in hepatic cells. Conversely, enhancing such enzymes in serum could also be used clinically as markers of liver injury [6]. In this investigation, DOX administration caused a significant increase in serum ALT, AST, and ALP levels. ALT is found in hepatocytes and is more susceptible to be released when the liver is injured. In clinical settings, modulation of liver enzymes and inflammatory factors with abnormal enzymatic contents are of paramount importance for diagnosis of liver damage [45]. In contrast, an increase in both ALT and ALP activities has also been found to be proportional to the extent of hepatic damage [6]. Supplementation of diosmin (100 and 200 mg/kg) caused a significant decrease in enzymatic activities and therefore induced a considerable preventive effect against hepatotoxicity [32]. These data demonstrated that DOX increased hepatic function serum ratio, including AST, ALT, and ALP levels. These effects were reported in an animal model involving DOX-induced hepatotoxic [46].

Antioxidant-mediated enzymes have been considered a first-line protective mechanism against ROS production in the living organism [47]. Nuclear factor erythroid 2 (NFE2)-related factor 2 (Nrf2) is one of the most important key regulatory elements that can regulate hundreds of different antioxidant proteins. Many antioxidant proteins, such as GPX, SOD, HO-1, and CAT, are activated by the transcription factor Nrf2. HO-1 is a key heme-degrading enzyme that also helps to maintain cell microenvironment homeostasis. Our findings demonstrated that gene expressions of SOD and HO-1 were downregulated in Dox-exposed animals as compared to control group, which was consistent with earlier reports [48,49]. Diosmin treatment, however, restored the observed alteration in SOD and HO-1 gene expression. Lipid peroxidation is a known indicator of oxidative stress [39]. Studies have reported an increase in MDA content in response to DOX administration [50,51]. In the current study, we demonstrated a significant decline in the liver antioxidant defense in DOX-administered animals. MDA contents in the liver of DOX-treated rats were significantly elevated. This suggests that DOX can form free radicals following oxidative damage to biological molecules and lipid peroxidation in the membrane [52]. In addition, pre-supplementation with diosmin at a dose of 100 and 200 mg/kg attenuated these effects. This is consistent with the findings of a previous study [53]. GSH is an endogenous molecule that acts as an antioxidant for the detoxication of ROS produced via external and internal stimuli required to maintain homeostasis necessary to support the normal functioning of cells [54]. Catalase, a CAT, is an antioxidant enzyme that has been known to decompose H_2_O_2_ into O_2_ and H_2_O. This reaction serves as a protective mechanism against ROS production [55]. This study showed a considerable decrease in GSH levels and CAT enzymatic activity in DOX-administered animals compared to control animals, consistent with previous studies [39,56]. Nevertheless, diosmin pretreatment ameliorated the alterations in GSH levels and CAT activity in the hepatic system, highlighting the protective effect and antioxidant potential of diosmin.

Oxidative stress has been investigated as a crucial mediator responsible for inducing apoptotic cascades [57]. Damage to the mitochondrial membrane has been critically linked to episodes of organ toxicity. These changes were shown to be a consequence of the overproduction of ROS, causing an activation of the intrinsic cascade in the apoptotic machinery [58]. It has been shown that impaired mitochondrial membrane can release cytochrome c from the mitochondria into the cytosol, which triggers apoptotic signaling involving the activation of caspase-9 [30]. This process may trigger the activation of other enzymes, such as caspase-3. Alternatively, Bax, which is considered a pro-apoptotic protein responsible for the durability of the membranous porosity, triggers the release of cytochrome c, leading to an intrinsic apoptosis-mediated activity in cells and/or tissues [30]. In contrast, Bcl-2 is an anti-apoptotic protein found mainly in the outer mitochondrial membrane. This protein can stabilize the mitochondrial integrity and inhibit cytochrome c release into the cytosol. Therefore, cellular survival predominantly maintains the Bax/Bcl-2 ratio [59]. In this study, DOX treatment caused a significant increase in Bax and cleaved caspase-3 expression and a decrease in the expression of Bcl-2 protein. These observations are in accordance with previous findings [60]. The altered expression of both pro- and anti-apoptotic proteins returned to the normal condition following diosmin supplementation.

DOX treatment inhibited PI3K/AKT pathway, as reported in previous findings [61]. Indeed, the stimulatory action of the PI3K/AKT pathway is crucial for oxidation-mediated cell resistance and apoptosis [62]. Our results demonstrated the link between the activated form of p-AKT and Bcl-2 up-regulation and subsequently downregulation of Bax expression. A previous study supported the action of p38 mitogen-activated protein kinase (MAPK) in DOX-induced toxicity [63]. p38 MAPK is a member of the MAPK superfamily composed of four different isoforms: p38α, p38β, p38γ, and p38δ. It has been shown that activation of p38 MAPK is implicated in DOX-triggered apoptosis. In this study, we showed that activation of p-p38 MAPK is a potent enhancer of ROS. In the current study, we showed that diosmin may reverse such enhanced levels of ROS against DOX insult, in line with previous work [64]. NF-κB is a nuclear transcription factor that plays a pivotal role in the pathophysiology of drug-induced hepatotoxicity [65]. In its inactive stage, it is more capable of making complexes with its inhibitors, IKα and IKβ. Upon activation, IKα or IKβ promotes phosphorylation of IKβ due to an insult or oxidative stress, leading to the release of NF-κB, enabling its translocation into the nucleus. Our results demonstrated significant activation of NF-κB in response to DOX administration, in accordance with previous findings [66]. Diosmin pretreatment, however, significantly inhibited the NF-κB- p65 activation cascade. These findings are also consistent with previous reports supporting the capability of a natural compound to hinder NF-κB activation [67]. Inflammation is a key player in the Dox-induced hepatotoxicity [33]. In the current study, we examined the genes involved in inflammation, such as TNF-α, IL-1β, IL-6, and iNOS. We found that Dox treatment showed upregulated expression of these genes. Nevertheless, these alterations in gene expression of TNF-α, IL-1β, IL-6, and iNOS were mitigated by diosmin treatment in a dose-dependent manner. These findings were in accordance with previously reported studies [33,68]. Furthermore, histological evaluation observed in DOX-treated rats revealed marked alterations in liver tissues, such as extensive hepatocytes necrosis with marked neutrophilic infiltration [69]. Alternatively, pretreatment with diosmin attenuated hepatic necrosis, neutrophilic infiltration, and other dysfunctions, in line with the previously published report [67].

## 5. Conclusions

**Figure 8:**
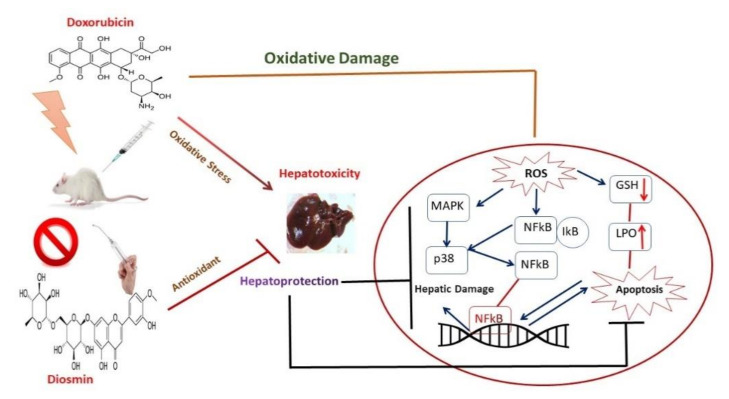
Schematic representation of hepatoprotective mechanism of diosmin against toxic effects of doxorubicin.

Data from the current study confirmed for the first time the hepatoprotective effect of diosmin against DOX-induced liver damage. Diosmin could be a potent regulator against DOX-induced liver injury. Our findings revealed that DOX significantly decreased hepatic antioxidant machinery, increased serum enzymatic activities, and disturbed the inflammatory and apoptotic-mediated signaling pathways. Importantly, diosmin pre-treatment restored DOX-induced oxidative stress and hepatotoxicity. The results support the implementation of diosmin for human consumption to fight against oxidative stress and its linked impairment. Further studies are necessary to fully understand the protective effects of diosmin against DOX-induced toxicity involving other potential signaling pathways. Further investigations should also establish an experimental schedule and/or formulation of such nutraceuticals against chemotherapeutics at other cellular and subcellular levels. The development of hepatoprotective compounds, including diosmin targeting the cellular compartment, delivers hope for future therapeutic interventions for various hepatic disorders (Figure 8). Nevertheless, one of the limitations of the present study is that we did not use inhibitors or knock-out models to inhibit the molecular mechanisms involved in doxorubicin-induced hepatotoxicity to validate the hepatoprotective effect of diosmin. However, the results of the current study shed light on the hepatoprotective effect of diosmin and would foster further studies to further prove the current findings.

## Figures and Tables

**Table 1:** List of primer sequences used in this study.

Gene	Accession Umber	Primer Sequences (5′→3′)	Product Length (bp)	Reference
SOD	NM_017050.1	Forward: TTCGTTTCCTGCGGCGGCTT Reverse: TTCAGCACGCACACGGCCTT	112	Custom-designed
HO-1	NM_012580.2	Forward: ACAGGGTGACAGAAGAGGCTAA Reverse: CTGTGAGGGACTCTGGTCTTTG	107	Custom-designed
IL-1β	NM_031512.2	Forward: CCAGGATGAGGACCCAAGCA Reverse: TCCCGACCATTGCTGTTTCC	519	Custom-designed
IL-6	NM_012589.2	Forward: GCCCTTCAGGAACAGCTATGA Reverse: TGTCAACAACATCAGTCCCAAGA	80	Custom-designed
iNOS	NM_012611.3	Forward: GGGAGCCAGAGCAGTACAAG Reverse: GGCTGGACTTCTCACTCTGC	138	Custom-designed
TNF-α	NM_012675.3	Forward: CACGCTCTTCTGTCTACTGA Reverse: GTACCACCAGTTGGTTGTCT	254	Custom-designed
GAPDH	NM_012675.3	Forward: TCTGCTCCTCCCTGTTCTAGAGACA Reverse: TTGTGAGGGAGATGCTCAGTGTTGG	1183	Custom-designed

## Data Availability

Data is contained within the article.
